# Humanising research on (non-)migration decision-making: a situated framework

**DOI:** 10.12688/openreseurope.16483.1

**Published:** 2023-09-07

**Authors:** Asuncion Fresnoza-Flot

**Affiliations:** 1 Laboratory of Anthropology of Contemporary Worlds (LAMC), Institute of Sociology, Faculty of Philosophy and Social Sciences, Universite libre de Bruxelles, Brussels, B-1050, Belgium

**Keywords:** humanising research, (non-)migration decision-making, thick contextualisation, life dimension-focused analysis, time-situated inquiry, societal drivers, engendering, decolonising

## Abstract

Recent global challenges such as the COVID-19 pandemic, economic crises, and wars have not impeded transnational migration to continuously unfold. The question of why some people migrate while others choose to stay remains one of the important preoccupations in migration studies. It underlines the need to further conceptualise transnational migration to identify the drivers behind individuals’ aspiration or intention to (re)migrate or stay where they are. Drawing from several migration theories and perspectives in various disciplines, this paper proposes the situated framework of “humanising research on (non-)migration decision-making”, that is, highlighting its human aspects. This scholarly enterprise is critically important as mainstream migration theories put more emphasis on individuals’ rationality, thereby overlooking other human aspects of migration and stasis. Viewing individuals as persons, this framework offers three ways to humanise the analysis: thick contextualisation, life dimensions-focused analysis, and time-situated inquiry. It also calls for the engendering of the analysis and decolonising the methodologies adopted in the study of (non-)migration decision-making.

## Plain language summary

This paper proposes an analytical framework to address the question of what drives people to migrate, remigrate, or stay where they are. To do so, it draws from existing migration theories in different disciplines and situates itself within the vast literature theorising migration. The resulting framework focuses on (non-)migration decision-making, specifically the drivers of migration aspiration and intention. It views individuals as persons with internal processes in cognitive, emotional, and relational terms; subjectivity; agency; social world; and lived experiences. This humanising framework not only calls for engendering research on (non-)migration decision-making but also suggests several decolonising data-gathering techniques. It offers three analytical ways: thick contextualisation, life dimensions-focused analysis, and time-situated inquiry. Its humanising approach to individual (non-)migration decision-making is a response to several calls to make scientific inquiries more humane, inclusive, and grounded.

## Introduction

Transnational migrations – the geographic movements of people across nation-state borders – have increasingly diversified in terms of the countries of origin, routes, destinations, and socio-demographic characteristics of the individuals involved ( Castles & Miller, 1993; Vertovec, 2007). The dynamic migration flows from non-traditional sending countries to non-conventional destinations are part and parcel of this phenomenon. The global COVID-19 pandemic, which started at the end of 2019, has affected the dynamics of transnational migrations, slowing it down by 27 per cent in 2020 ( UN DESA, 2019). Despite this effect, the overall population of transnational migrants has continued to rise: from 272 million in 2019 to 281 million in 2020 ( McAuliffe & Triandafyllidou, 2021, 3). The way in which this fast-growing phenomenon has been unfolding despite global challenges underlines the need to further conceptualise transnational migration to fully understand why some people move and others not.

Several decades of theorisation have produced a wide array of analytical frameworks that aim to uncover what drives people to migrate, remigrate, or stay where they are. Mainstream migration theories focus on individuals’ rationality to uncover the logics behind their spatial mobility, thereby neglecting other important dimensions of human lives. Consequently, such studies can only grab a fraction of the whole picture and miss equally significant triggers to migration. To capture the whole picture, the present paper delves into individuals’ (non-)migration decision-making and proposes a framework that humanises research on this theme, that is, highlighting the human aspects of individual decision-makers. The term “(non-)migration decision-making” refers to the process during which individuals “come to a decision not just as after-the-fact listing of good or rational reasons” ( Koikkalainen & Kyle, 2016, 759) to migrate or not. It is employed here to recognise that an individual’s decision to move can change over time. Aware that decision-making is a long and fluid process that is susceptible to changes, this paper pays attention to both voluntary and “involuntary immobility” ( Carling, 2002) in which people aspiring to move may not be able to do so due to the lack of mobility options ( De Haas, 2021). In other words, it considers migration and immobility as part of a socially, temporally, and psychologically situated continuum.

This paper builds its proposed framework by drawing from existing migration theories and perspectives in different disciplines, namely sociology, anthropology, geography, and psychology. By doing so, it situates the framework within the vast literature of migration theorisations to which it intends to contribute fresh insights. The resulting framework focuses on the “drivers” of migration aspiration and intention. “Drivers” pertain to the “external material forces that influence mobility” ( Van Hear *et al.*, 2018, 928) and to the internal and relational processes in which individuals are enmeshed. The term “aspiration” is understood in this paper as one’s “wish” to migrate or not, whereas “intention” means the individual’s “preparation to migrate” or, in short, the “ultimate step of an individual migration project” ( Migali & Scipioni, 2019, 182). Including intention to migrate in the paper’s proposed framework can allow scholars to “capture future migrants” (ibid., 192) in their respective studies and understand the “involuntary immobility” ( Carling, 2002) of others. Heeding the recent call to include time in the analysis of migration ( Griffiths *et al.*, 2013; King *et al.*, 2006), this paper integrates the time dimension in its analytical framework.

Before introducing its proposed framework to humanise research on (non-)migration decision-making, the paper starts by revisiting the existing theories of migration. This exercise aims to identify the lacunae in migration theorisations, which provides the ground from which to build its analytical framework. The core of the paper presents the constitutive elements of the framework: its decolonising and engendering approaches as well as its concrete analytical ways to emphasise the human aspects of (non-)migration decision-making. The paper also suggests some methodological directions in how to pursue humanised research on the topic. It ends with reflections on the scientific strengths and possible social impact of the framework.

## Revisiting theories of migration

Since the advent of migration studies, scholars in different disciplines have explained human spatial mobility in many ways. The frameworks they crafted can be generally understood based on their disciplinary embeddedness, dimensional orientation, level of analysis, and the way they perceive migration.

**Figure 1.**
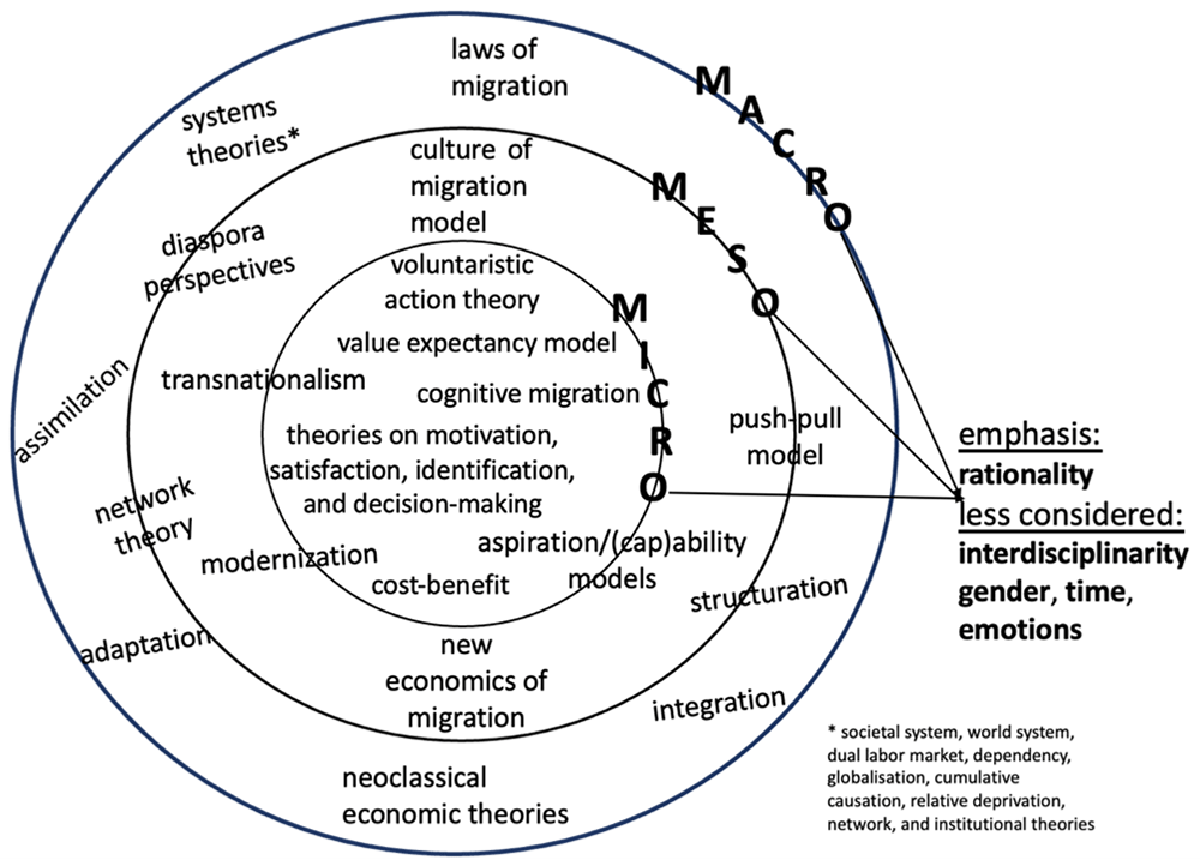
State of mainstream migration theorisations

In terms of disciplinary embeddedness, widely utilised migration theories as shown in Figure 1 have been designed in the field of economics: for example, the relative deprivation theory ( Stark, 1984), neoclassical economics ( Todaro, 1989), and the dual labour market theory ( Pryor, 1979), among others. These theories emphasise the economic benefits that migration brings to households, communities, and nations. They explain migration as resulting from a disequilibrium between labour demand and supply and underline the rationality of individuals. Their fixed focus on the economic aspects has been criticised by scholars for neglecting the non-economic determinants of migration and the individual’s “agency” – the ability “to make independent choices and to impose these on the world and, hence, to alter the structures that shape and constrain people’s opportunities or freedoms” ( De Haas, 2021, 14). Theories of migration in other disciplines provide alternative lenses. For instance, sociological theories go beyond the economics of migration ( Richmond, 1988) by scrutinising the impact of societal changes ( Massey, 1990), social networks/capital ( Choldin, 1999; Massey *et al.*, 1998), and global systems on the individual’s migration decision ( Sassen, 1988; Wallerstein, 1974). Geographical theories explain migratory phenomena through the analysis of spatial patterns of human mobility ( Ravenstein, 1885), as well as the individual’s aspiration (i.e., wish to move or stay) and ability to move ( Carling, 2002; Carling & Collins, 2018) situated in its social contexts. Anthropological theories pay attention to multifaceted social links, power asymmetries in the local and transnational arena, as well as tangled forms of mobilities ( Glick Schiller *et al.*, 1992; Fresnoza-Flot & Liu-Farrer, 2022; Mahler & Pessar, 2001; see also the reviews by Brettell, 2000 and Horevitz, 2009). These frameworks encompass different levels of analysis, but many of them pay limited attention to the temporal and psychological dimensions of individual’s migration decision-making.

Regarding dimensional orientation, migration theories focus mostly on the role of social networks and the impact of the individual’s immediate entourage on his/her migration decision. Concerning the latter, individuals who have children or ageing parents may opt to stay in their country of birth or, if they are migrants, they may decide to return to their country of origin ( Achenbach, 2017). The rise of the “Gender and Migration” scholarship has reinforced this focus on the relational dimension of migration by critically analysing power relations in which migrants and non-migrants, women and men, as well as children and parents, are involved in the realm of home and workplace. This engendering of migration studies has accompanied the emergence of gender-focused migration theories. The term “gender” refers to a socially constructed institution that prescribes roles and behaviour to men and women ( Lorber, 1994). Gender can also be considered as a process that requires “performance” ( Butler, 1990). For example, the perspectives of the “new international division of reproductive labour” ( Parreñas, 2000), the “global care chains” ( Hochschild, 2000), the “feminisation of survival” ( Sassen, 2000), and the “gender geographies of power” ( Mahler & Pessar, 2001) have all helped scholars unveil the (re)production of unequal power relations in gender terms in which migrants (notably women) experience and participate. These theories have emerged at the same time as others focusing on “transnational families” and households ( Bryceson & Vuorela, 2002; Le Gall, 2005), in which members are physically separated from one another due to migration but maintain a sense of solidarity across national borders ( Baldassar & Merla, 2014). This specific literature on transnational families/households documents the way in which gender norms and expectations, as well as the normative ideals of “good” mothering and fathering, influence an individual’s decision to migrate or stay. Despite the rich literature on families/households and gender and migration, gender-focused theories remain at the fringe of the mainstream migration theorisations and are most often forgotten in reviews of analytical frameworks explaining migration. Mainstream migration theories continue to be either gender neutral or to treat gender as a variable like social class, age, ethnicity, and nationality.

Another dimension most often overlooked in existing theories of migration concerns emotions and other cognitive processes, which is partly due to the scholarly emphasis on an individual’s rationality during migration decision-making. The emotional process involves feelings such as fear, sadness, and guilt, whereas the cognitive process refers to individual’s imaginaries, memories, and “cognitive map” ( Gärling & Golledge, 2000). For the last few years, certain theories and empirical works have pointed out how imaginaries and emotions affect an individual’s decision-making process ( Boccagni & Baldassar, 2015; Carling & Collins, 2018; Wang & Chen, 2021). Despite this development, the internal processes (cognitive and emotional) of individuals remain rarely treated together in the analysis. In addition, scholars increasingly criticise the neglect of the temporal dimension in the analysis of migration ( Griffiths *et al.*, 2013). A few studies that do so locate migration decision-making within the past and present situations, which underplays the future and the change(s) that may arise for aspiring (re)migrants.

As regards level of analysis (see Figure 1), macro-level migration theories unravel the socio-political and economic forces driving migration and identify the demographic characteristics of migrants ( Pryor, 1981; Richmond, 1988). These theories have been criticised for neglecting individual agency. Meso-level migration theories have examined so far the role of social networks and ties, specifically social capital ( Bourdieu, 1986) based on kinship, household, ethnic, and diaspora affiliations, among others ( Boyd, 1989; Faist, 2020; Massey, 1988; Van Praag *et al.*, 2021). This focus overlooks the structuring influence of larger societal forces such as restrictive migration policies on an individual’s choice of which social ties to tap into, reinforce, or set aside during migration decision-making. Contrary to macro- and meso-level theories, micro-level analytical frameworks underline individual agency, psychological factors, and social identities (e.g., gender, age, social class) to explain migration. Their individual-focused analysis is often viewed as insufficient to capture macro- and meso-level structuring factors such as the impact of gender norms and social networks on migration decisions. Whereas macro-level theories have been widely adopted in migration studies for their generalising power, meso- and micro-level frameworks, specifically their combination and articulation with macro-level lenses, remain scarce (e.g., Carling’s aspiration/ability framework and De Haas’ aspiration/capability model).

Finally, based on how they view migration, theories can be functionalist, historical-structural, or agency-focused ( De Haas, 2021). Functionalist theories perceive migration “as a way to create more equality within and between countries”, whereas historical-structural theories see migration “as a way to maintain and reinforce existing inequalities between and within countries” ( Van Praag *et al.*, 2021, 19; see also Morawska, 2015). These conventional perspectives fail to fully grasp how individuals and groups “exert agency within broader structural constraints” ( De Haas, 2021, 14), a limitation that agency-focused theories address. This latter set of theories highlights micro- and meso-level factors by examining individual social networks at local, transnational, and diasporic spaces (e.g., Faist, 2004); migration systems ( Massey, 1988); and culture of migration ( Timmerman *et al*., 2014), among others (e.g., Van Praag *et al.*, 2021). Nonetheless, in their analysis, these theories do not pay sufficient attention to the power of emotions and cognitive processes.

The above review highlights the major lacunae in migration theories, which Figure 1 above summarizes. First, there is the need for migration theorisation to be more interdisciplinary ( Fresnoza-Flot, 2022; Koikkalainen & Kyle, 2016; Hondagneu-Sotelo, 2011) as each discipline offers a migration theory suited for a specific context and situation. Except for the aspiration/capability model ( De Haas, 2021), which adopts an interdisciplinary approach, theories attempting to explain migration remain within their respective discipline. Second, each theory focuses either on one level or two levels of analysis but very rarely articulates the micro and meso levels with the macro one. When the three levels are taken into account, scholars most often examine them separately, during which some nuances of the analysed data may elude their critical gaze. Third, multidimensional perspective appears less attractive than unidimensional approach. Rationality persists in being central to the analysis, which overlooks other life dimensions, notably internal processes (emotional and cognitive), time (stages and timing), and individual’s social locations “within interconnected power hierarchies” ( Pessar & Mahler, 2003, 816). To sum up, the lacunae above suggest the critical importance of interdisciplinarity, multilevel perspectives, and multidimensionality in theorising migration. When considered together, these epistemological stances form a framework highlighting the human aspects of individuals’ (non-)migration decision-making as the next section unveils.

## Framing (non-)migration decision-making using a humanising lens

By adopting an interdisciplinary, multidimensional stance based on multilevel perspectives this paper provides a framework that views individuals as persons with internal processes in cognitive, emotional, and relational terms; subjectivity or sense of self; agency; social world; and lived experiences.

The proposed framework is called here “humanising (non-)migration decision-making” (see Figure 5) for three reasons. First, it avoids a dichotomic approach by considering rationality and emotionality as part of the same internal process of individuals. Rationality and emotionality are mutually reinforcing psychological mechanisms and favouring one over the other in the analysis de-humanises individuals. Second, the proposed framework underlines that the individuals’ decision to migrate or not is a “social fact” ( Durkheim, 1894), reflecting not only the psychological processes they underwent and/or are experiencing but also the broader structural situations in which they are enmeshed. It emphasises the importance of identifying from different angles the drivers of individuals’ aspiration or intention to migrate. Third, building from gender and migration scholarship, it calls for the “engendering” ( Mahler & Pessar, 2006) of the mainstream migration theories in which gender remains at the fringe. It recognises the importance of gender in the study of migration decision-making to deeply understand what drives people to migrate or to stay. To highlight the human aspects of (non-)migration decision-making, it offers three concrete analytical ways: thick contextualisation, life dimensions-focused analysis, and time-situated inquiry.

### Thick contextualisation

**Figure 2.**
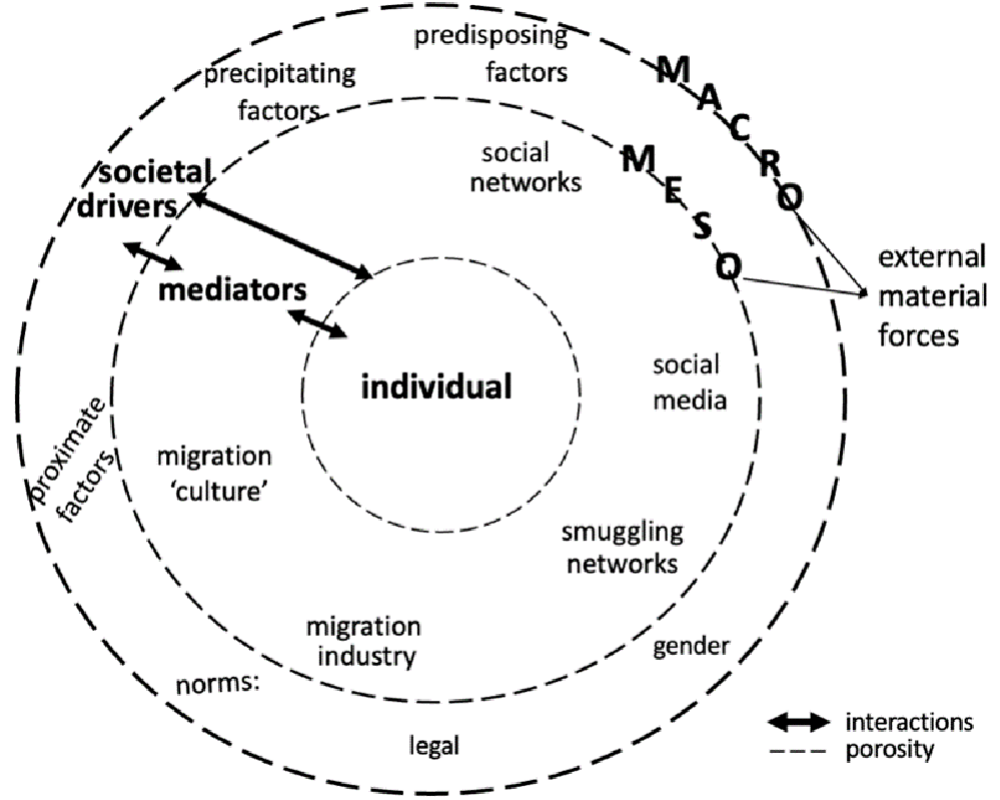
Thick contextualisation of individual aspiring to (re)migrate or stay

Since “macro-level factors” can shape “the contexts that affect meso- and micro-level factors” ( Van Praag *et al.*, 2021, 28), it is crucial to grasp what factors are through thick contextualisation. Inspired by Geertz’s (1973) “thick description”, thick contextualisation means providing detailed information about the specific social world an individual inhabits. It implies paying critical attention to societal drivers (see Figure 2) that motivate individuals to migrate or to stay.

Societal drivers are akin to what Van Hear and colleagues call “external material forces” and can take four forms: “predisposing” (structural gaps between two countries stemming from the “global macro-political economy”), “proximate” (factors emanating from “deep-seated structural features”), “mediating” (meso-level factors that “enable, facilitate, constrain, accelerate”, consolidate or diminish migration), and “precipitating” (“identifiable event or events” directly affecting families and households) ( 2018, 931–932).

At the macro level, predisposing (e.g., economic disparity between countries) and proximate factors (e.g., effects of climate change and new employment opportunities) appear to be powerful societal drivers. For example, the disparity in terms of affordability between healthcare services in Thailand and those in foreign countries drive Western retirees to migrate to the former ( Sunanta & Jaisuekun, 2022). Likewise, employment opportunities drive people to move first across transnational and then local borders ( Ng, 2023). Other important societal drivers are legal and gender norms. Legal norms concern “regimes of mobility” ( Glick Schiller & Salazar, 2013) in the country of residence of aspiring re/migrants and their desired country of destination. They are the reflections and direct results of states’ “governmentality”, that is, the management of their people through “institutions, procedures, analyses and reflection” ( Foucault, 1991, 102). Legal norms in the form of state policies on human spatial mobility are an instrument to manage, regulate, and control people’s movements. These norms influence individuals’ strategies to attain their migration project for themselves and/or for reuniting family members through the help of membership intermediaries ( Bonizzoni & Fresnoza-Flot, 2023). For example, mobility policies with strong social class or economic capital criteria incite elite migratory movement, such as that of people with Chinese citizenship in Portugal thanks to the golden visa scheme in this country ( Gaspar & Ampudia de Haro, 2020). Since gender is part and parcel of broader social processes, gender roles and expectations on men and women in the family and larger society should be taken into account as they may motivate these individuals to migrate or to stay. For instance, whereas Vietnamese parents migrate “ *because* of their kids” ( Souralová, 2014, 175), Filipino lone mothers leave abroad to be “good” mothers to their children ( Asis & Ruiz-Marave, 2013). Gender ideology – “a set of attitudes about the appropriate roles, rights, and responsibilities of men and women in a given society” ( Lucas-Thompson & Goldberg, 2015, 13) – shaped by religion and/or other dominant societal perspectives may also act as a strong driver for individuals’ migration aspiration. At the meso level, gender ideology mediates factors that are essential societal drivers. They have various forms, namely social media, smuggling networks, migration culture, and the migration industry. These factors provide individuals with information about possible ways to migrate and to reach their destination country. At the micro level, precipitating drivers (e.g., insufficient welfare services, economic crisis, sudden unemployment) can directly affect individuals’ well-being and motivate them to aspire to migrate.

Figure 2 illustrates the articulation of the micro- (the individual), meso-, and macro-level structural factors with one another, which can reveal the porosity of the supposed boundaries between them. Thick contextualisation may be descriptive, but it is indispensable to comprehend what an individual undergoes in psychological and relational terms when thinking, imagining, feeling, or planning to migrate.

### Life dimension-focused analysis

**Figure 3.**
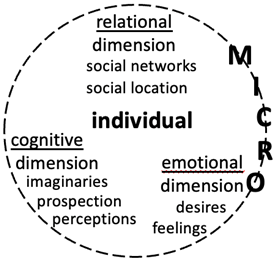
Internal processes at the micro level

The framework “humanising (non-)migration decision-making” entails a rigorous analysis of individuals’ internal processes, that is, their different cognitive, emotional, and relational life dimensions as shown in Figure 3. It can show that rationality and emotions are mutually reinforcing dimensions and that social relations play a key role in decision-making.

To examine the cognitive dimension, it is important to build on several perspectives exploring the mind and consciousness. Koikkalainen and Kyle’s “cognitive migration” framework that underlines the “role of imagination and prospective thinking in migration decision-making” ( 2016, 769) appears useful in this regard. Two other frameworks offer innovative insights: first “legal consciousness”, which unveils how ordinary people view the law and talk about it in their everyday lives ( Ewick & Silbey, 1998); and second, “transnational consciousness”, which uncovers migrant’s “abstract awareness of one’s self, diaspora and multiple belonging” ( Ghosh & Wang, 2003, 278). These frameworks bring attention to individuals’ imaginaries, that is, their mental images, visions, ideas, thoughts, stereotypes, fantasies, memories, and clichés ( Salazar & Graburn, 2014). They also emphasise the need to consider individuals’ prospective thinking, notably the way they view and plan the future. Perceptions of macro (cultural, historical, economic, political, and environmental) and meso structural factors, as well as their embeddedness in broader social networks, can help pinpoint which societal drivers play an active or passive role in individuals’ (non-)migration decision-making.

As regards the emotional dimension, the fast-growing literature on emotion and migration highlights the importance of focusing on individuals’ desires and feelings (e.g., guilt, fear, sadness, excitement, satisfaction, dissatisfaction) as they envision and imagine migrating or staying. For instance, what impede Asian highly skilled migrants to leave Japan are their affective and social ties developed from staying longer in the country and/or marrying a Japanese citizen ( Liu-Farrer, 2023). By considering emotions, the analysis avoids over-simplification and brings the psychology of migration directly in dialogue with existing migration scholarships in which migrants and individuals aspiring to migrate or stay are viewed as rational actors.

Concerning the relational dimension of an individual’s psychology, the privileged focus is on interpersonal ties that individuals view, consider, or believe as composing their social universe. This dimension encompasses the realm of the family and household, the public realm where individuals construct social ties, and the work environment ( Achenbach, 2017). Drawing from the aspiration/ability framework ( Carling & Collins, 2018) and the aspiration/capability model ( De Haas, 2021), this paper accentuates the need to scrutinise how individuals’ local and transnational social networks (family, household, community, friendship, migrant traffickers) affect their ability/capability to aspire to migrate or not. Social networks are important to include in the analysis as they shape people’s imaginaries and expectations regarding migration ( Hernández-Carretero & Carling, 2012; see also Ryan, 2023).

In addition, it should be noted that the relational dimension of human life is amplified by interpersonal interactions, during which individuals’ social locations (i.e., different social identities based on gender, social class, age, and so on) intersect with one another, making it easy or difficult to “access […] resources and mobility” ( Pessar & Mahler, 2003, 817). This articulation of various social identities is important to understand as they shape, discipline, and position individuals within power hierarchies ( Mahler & Pessar, 2001). In this case, Crenshaw’s “intersectionality” ( 1989) perspective appears useful to highlight the social mechanisms behind the (non-)migration decision-making of social minorities, notably women. This perspective unpacks how the simultaneous interaction of social identities (e.g., gender, social class, age, among others) (re)creates individuals’ marginality and precarity.

The internal processes described above and exposed in Figure 3 need to be thickly contextualised in order to grasp their dynamics and the factors shaping them. Nonetheless, locating them within macro- and meso-level social situations appears incomplete without situating them in time that shapes cognitive and emotional processes during decision-making.

### Time-situated inquiry

**Figure 4.**
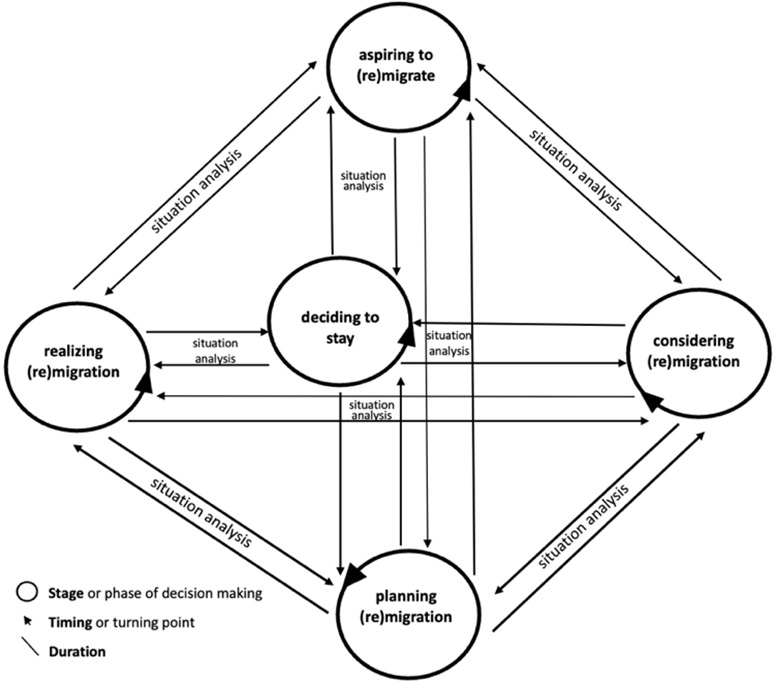
Temporality of migration (non-)migration decision-making inspired from existing models

Drawing from time-sensitive studies on (non-)migration decision-making ( Achenbach, 2017; Griffiths *et al.*, 2013; Kley, 2011; Van der Velde & van Naerssen, 2011), the proposed framework in this paper focuses on two aspects: the stages of (non-)migration decision-making, and its timing. Whereas stages refer to the evolution over time or the successive phases of an individual’s aspiration and/or intention to (re)migrate or not, timing refers to the turning points or the specific moment(s) of change(s) in individuals’ decision about whether to (re)migrate or stay. Given the unpredictability of individuals’ behaviour, these temporal aspects of decision-making take place in a circular way, as Figure 4 shows, rather than in a linear fashion.

**Figure 5.**
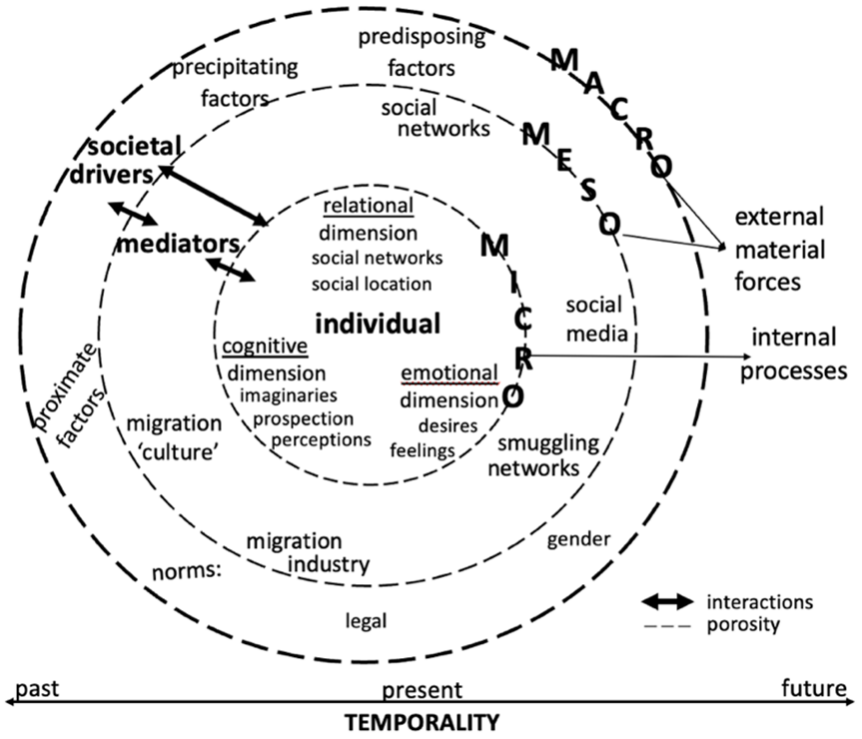
The “humanising (non-)migration decision-making” framework

Decision-making may start with a wishful thinking of migrating, or in other words, “aspiring to migrate”. After this stage, the individual may embark on “situation analysis” ( Achenbach, 2017), weighing up the pros and cons of as well as the resources available for (re)moving or staying. The result of this analysis may either be the state of “considering migration” ( Kley, 2011) (i.e., “mental threshold” or individual’s mindset: Van der Velde & van Naerssen, 2011) or the decision not to pursue migration. This stage may also include a “locational threshold” ( Van der Velde & van Naerssen, 2011), during which an individual chooses a destination country. Once an option is chosen, the next stage may be the evaluation (i.e., situation analysis) of such a choice ( Achenbach, 2017). If the final choice is migration, the individual concerned may embark on “planning migration” ( Kley, 2011) or a “trajectory threshold” ( Van der Velde & van Naerssen, 2011), during which specific migration routes are identified to reach the target destination. This stage can also be called the phase of “preparation to migrate” ( Migali & Scipioni, 2019). The final stage of decision-making would be the act itself of migrating, i.e., “realizing migration” ( Kley, 2011). These stages appear limited as they may overlook the “on-going complex and often opportunistic rather than planned” individual’s decision-making ( Griffiths *et al.*, 2013, 16). They may neglect intermediary phases during which several temporalities may arise, such as “waiting” and “being still” (ibid.).

To address this limitation and to capture all the stages of the decision-making process of aspiring (re)migrants, it is essential to consider the timing of the individual’s aspiration and/or intention that is shaped by his/her past life, present situation, and imagined or desired future ( McCormack & Schwanen, 2011). Since individuals experience time differently depending on their social locations, social capital, and available financial resources, the duration of each stage and intermediary step of their (non-)migration decision-making may vary from one moment to another. Duration also refers to the time frame of a particular external driver of migration (see Figure 4), such as political crisis and economic turmoil ( Van Hear *et al.*, 2018), and the way in which individuals experience such a duration is important to note to understand the link between time and the aspiration/intention to move or not. Besides, mobility involves discontinuities or ruptures not only in emotional terms but also temporalities (see Korpela, 2023).

A time-situated analysis can facilitate the identification of the specific stage in which societal drivers such as spatial mobility policies of the target country of destination matter in the decision-making of aspiring (re)migrants. As Figure 5 suggests, it is an integral part of a humanising approach to migration decision-making as it provides an interesting ground on which thick contextualization and life dimensions-focused analysis can be fully carried out.

## Humanising methodologies

The framework “humanising (non-)migration decision-making” aims at “decolonizing” ( Lincoln & Gonzalez y Gonzalez, 2008; Smith, 2021) methodological approaches by putting emphasis on emic perspectives (i.e., individual’s points of view) and diversity of voices, discourses, and experiences. Decolonisation as a “psychological project” ( Bhatia, 2020) involves disruption of conventional research approaches to embrace ethical, reflexive, and empirically grounded ways of knowledge production in which the voices of marginalised people are valorised and those of the socially visible are decentred ( Smith, 2021). In other words, it brings to the fore “vernacular knowledge”, that is, mostly “orally transmitted knowledge” ( Tilley, 2010, 112). In the context of research, this form of knowledge can be wholly captured through a triangulation approach combining qualitative and participatory methodologies.

Whereas qualitative methods value the depth and critical analysis of empirical data, participatory methods include the active involvement of study participants in data collection, thereby promoting the co-production of knowledge. Both methods promote participants’ voices and agency, allowing the researchers to capture aspiring individuals’ decision dynamics regarding (re)migration and stasis. There are several possible data collection techniques that can be qualified as qualitative and/or participatory. Considering the analytical ways of humanising research on (non-)migration decision-making exposed in the previous section, this paper provides some examples of techniques that can highlight the human aspects of (non-)migration decision-making.

To pursue thick contextualisation of aspiring or intending migrants’ decision-making, researchers need to provide detailed information about the social world these individuals live in. This means exploring the possible pertinent contexts of their social world: for instance, its (colonial or post-colonial) historical embeddedness and its “external material forces” in all their forms ( Van Hear *et al.*, 2018). Aside from the social world, the natural environmental contexts should be given critical attention as they also strongly shape individuals’ everyday lives. Archival and other forms of documentary research, as well as content analysis of selected documents (e.g., texts of laws and policies, historical accounts, journalistic and government reports, or statistical data), appear heuristic approaches to produce thick data and a solid analysis. Nonetheless, having thick data at hand is insufficient if they are not empirically grounded. This means establishing a link between the empirical data collected from study participants and the contextual data at hand. Doing so allows researchers to determine which specific contextual data are pertinent to and should be highlighted in their respective studies. The empirical data that will be used as the ground of contextualisation can be obtained in several ways. One example is the participatory technique called “focus group discussion” ( Seal *et al.*, 1998), during which researchers provide the setting for a dynamic dialogue around specific topics among informed consenting participants. Another data collection technique that can yield rich insights is to conduct individual semi-structured interviews revolving around issues concerning migration or non-migration decisions.

This technique is also effective in gathering data about different life dimensions of aspiring or intending migrants, allowing to obtain data about the reasons behind their migration aspiration and to generate narratives replete with emotions. Regarding the relational dimension of human lives, although semi-structured interviews can gain information about aspiring migrants’ social networks, the technique called “social network analysis” ( Froehlich *et al.*, 2020) remains to date the most widely employed method. It can be carried out in many ways: for example, through “concentric circles method” ( Van Waes & Van den Bossche, 2020) or through participatory social-network mapping using online tools or specific software.

To situate (non-)migration decision-making in its temporality effectively, a life course perspective ( Kley, 2011) relying on biographical interviews appears useful. Nonetheless, since this data collection technique is mostly retrospective and much less prospective, it can benefit from integrating a prospective dimension. Biographical-prospective interviews can capture the intersecting effects of an individual’s (non-)migration decision past, present, and imagined future. These interviews yield insights into both retrospection and “prospection” ( Seligman *et al.*, 2013). Such an approach allows researchers to identify the different emotions, imaginations, and expectations that come out during each narrative of study participants and, most importantly, to determine at what stage of the decision-making process these individuals are situated: at the beginning when they are aspiring to move, in the process of preparing their voyage, or about to change their decision? Another data gathering technique that can capture the temporality of (non-)migration decision-making is the collection of solicited diaries – “diaries that people have been asked to keep for a particular reason, notably for research purposes” ( Bartlett & Milligan, 2021, 3). They can be in one of the following formats: written ( Rauch & Ansari, 2022), audio ( Monrouxe, 2009), video ( Zundel *et al.*, 2018), or photographic ( Swallow *et al.*, 2015). Such technique allows researchers to observe the evolution of participants’ migration aspiration over a given period of time, specifically the transition from one stage to another and the timing of their decision-making. It can be short-term (e.g., during one year) or longitudinal, spanning several years. It can also unveil the stage(s) during which specific societal drivers and mediators affect or influence the participants’ decision to move or to stay.

Like other qualitative and participatory methods, the data collection techniques described above, when adopted, require dynamic reflexivity of the researchers to be aware of and to try to reduce inequalities between them and the participants during the research process. As Smith argues in her work *Decolonizing methodologies* ( 2021), researchers working with marginalised groups need to “pay particular attention to matters that impact on the integrity of research and the researcher, continuously develop their understandings of ethics and community sensibilities, and critically examine their research practices” (261).

## Conclusion

This paper proposes a framework that aims to humanise research on (non-)migration decision-making. Its starting analytical point is the individual who is aspiring or intending to migrate, remigrate, or stay. To capture the dynamics of individuals’ (non-)migration decision-making, it underlines the importance of viewing individuals as persons embedded in their social world and with cognitive and emotional processes, as well as multiple social links. The strengths of the framework lie in its analytical and methodological contributions, as well as in its possible social impact.

In analytical terms, unlike most mainstream migration theories, the framework advanced in this paper adopts an interdisciplinary, multi-level perspective, and multidimensional posture. It does so by building from different theories and perspectives on migration in various disciplines; by articulating the micro-, the meso-, and macro-level structural factors with one another; and by considering the rationality, emotions, and relational dimension of aspiring migrants. Through its epistemological stances, the framework offers three concrete ways to humanise research on (non-)migration decision-making, which will allow scholars to identify the specific drivers of individuals’ aspiration or intention to migrate or not. First, it calls for thick contextualisation of the individuals’ lives by inquiring into the characteristics of their social world and by empirically grounding this inquiry by putting emphasis on the link between individuals’ lived experiences and the contexts they live in. Second, it encourages an analysis focused on life dimensions by delving into individuals’ internal processes in cognitive and emotional terms and their social relations while paying a critical attention to their intersecting identities such as gender, social class, and other parameters. And third, it promotes time-situated inquiry by paying attention to the stages and timing of (non-)migration decision-making, which remain largely neglected in mainstream migration theories. Through these analytical ways, the framework proposed in this paper provides a holistic approach to the study of (non-)migration decision-making, highlighting an individual’s agency and situating it in broader social contexts and temporalities. Across the analysis, it also integrates a gender approach, specifically calling for the “engendering” ( Mahler & Pessar, 2006) of the study at the macro, meso, and micro levels.

Regarding its methodological contributions, in line with its engendering approach and inspired from “decolonising methodologies” ( Smith, 2021), the framework brings further to the fore what qualitative researchers and critical feminist scholars have been doing – valorising the voices and perspectives of socially invisible groups. It does so by endorsing a triangulation approach combining several qualitative and participatory data collection techniques, which can facilitate step-by-step thick contextualisation, life dimension-focused analysis, and time-situated inquiry. It prioritizes methodological techniques that bring out the human aspects of individuals by treating them wholly as persons with rationality, emotions, and changing behaviour situated in time and social contexts. It also emphasises the importance of researchers’ reflexivity when engaging in humanising methodologies to ensure ethical and respectful knowledge (co-)production.

Considering its scientific contributions, the framework proposed in this paper will be particularly useful to studies inquiring into the causes of a specific migratory phenomenon (such as migration or remigration) and aiming to influence policymaking. Its holistic approach to individual (non-)migration decision-making is a response to several calls to make scientific inquiries more humane, inclusive, and grounded. A human approach to (non-)migration decision-making is critically important to understand not only migration dynamics but also voluntary and “involuntary immobility” ( Carling, 2002) against the backdrop of nation-states’ control of transnational migrations in which individuals are often treated as void of “bare life” ( Agamben, 1998). When treated as filled with life, these individuals are “primarily considered as either productive workers (with skills of varying desirability in different sectors of the economy) or reproductive laborers (with character traits suitable for marriage partners)” void of emotions ( Liu-Farrer & Fresnoza-Flot, 2022, 252). The insights resulting from the use of the framework proposed here will inform policymakers and migration agents on the indispensability of viewing and treating migrants not just as rational, objective persons but also as emotionally sensitive social beings.

## Ethics and consent

Ethical approval and consent were not required.

## Data Availability

No data are associated with this essay as it is the fruit of the author’s analysis of existing literature.
