# Supplementary material for: Humanising research on migration decision-making: a situated framework
Source: Open Res Eur. 2024 Feb 13;3:142. Originally published 2023 Sep 7. [Version 2] doi: 10.12688/openreseurope.16483.2 (PMC11109685; doi:10.12688/openreseurope.16483.2)
Supplement: Peer review history

# Reviewer report: Reviewer response for version 2

**DOI:** 10.21956/openreseurope.18656.r47981

**Published:** 2025-01-21

**Authors:** Nils Witte

**Affiliations:** 1 Institute for Sociology, Goethe University Frankfurt, Frankfurt, Frankfurt, Germany; 2 Federal Institute for Population Research, Wiesbaden, Germany

The manuscript has been submitted for the format “essay”. According to the ORE-guidelines “essays are analytic or interpretive compositions on a single topic. This could include articles outlining an argument or personal point of view.” The author describes the format as a review essay, a format that ORE does not define. What the manuscript does, however, is to develop a holistic theoretical model of (non)-migration decision making. The theoretical model is combined with instructions of how to do empirical research in order to live up to the moral and scientific goals of this model. So that fits the category offered by ORE of “outlining and argument”. So, I will evaluate whether the argument is coherent and convincing.

 The ambition of the essay is laudable, an encompassing theory of transnational migration would be a welcome contribution to migration research. Even if a general theory cannot be achieved, it is important to question existing theories and reflect how they can be complemented and improved. In its current form, however, there seems to be too much antagonization of existing migration theories and empirical migration research. Strategically, it might be a better alternative to frame the endeavour as a synthesis of existing theories. The synthesis can then be improved by the contributions proposed by the author.

 My concerns have been largely addressed by previous reviewers of this manuscript and its earlier version. My impression is that the author does not intend to substantially overhaul the structure or argument of the manuscript. Instead she has successfully taken the comments as an opportunity to improve the manuscript in its current form. That is fine but I will therefore, not repeat all of the previous reviewers’ comments.

 The author has already responded to the concern that the claim of “humanizing migration research” comes across as moralizing and a critique of previous research on migration research. I have the impression that this notion remains. The abstract suggests that classical theories of migration dehumanize migrants by suggesting rationality. One could hold against that that rationality is a human characteristic and that there are various theories in the “classical” canon and some include ideas of bounded rationality, sociality, and households as decision making units. Each one of these approaches has their merits and their weaknesses. My feeling is that the model promoted in this essay could be made more acceptable to readers if it acknowledged the contribution of (classical) migration theories and softened the moral judgement.

 As a theory, the outlined model aims to humanize research subjects and give them a voice and decolonize migration research. Less holistic theoretical models might not achieve these same goals, but they have other strengths including parsimony and the ability to generate testable claims and generalize beyond individuals as persons (to take the other extreme, which seems to be favored in this essay). Depending on the research question and the scope of the study, one or the other approach will be more fitting to achieve the research goals.

 What is the value added of emotions and cognitive processes in migration decision making? In one of the examples given to underline the significance this boils down to being married to a national of the residence country or feeling attached to it. This is conveniently integrated in quantitative surveys and modelled by existing migration theories. Maybe the author finds other examples of emotions and cognitive processes that illustrate why we need a new theoretical model.

 Analytical precision remains an issue. The text contains some tautological statements (e.g., "Perceptions of macro (cultural, historical, economic,

 political, and environmental) and meso structural factors, as well as their embeddedness in broader social networks, can help pinpoint which societal drivers play an active or passive role in individuals’ (non-)migration decision-making").

 While the conclusion claims that the model will help identify causes of migratory phenomena, the figures suggest that elements of the theory mutually cause each other or interact (in particular Figure 4). The explanatory value would be higher if the assumed relations were more specific.

Is the argument persuasive and supported by appropriate evidence?

Partly

Is the work clearly and cogently presented?

Partly

Is the topic of the essay discussed accurately in the context of the current literature?

Partly

Does the essay contribute to the cultural, historical, social understanding of the field?

Yes

Reviewer Expertise:

skilled migration; migration from the Global North; crisis migration

I confirm that I have read this submission and believe that I have an appropriate level of expertise to confirm that it is of an acceptable scientific standard, however I have significant reservations, as outlined above.

# Reviewer report: Reviewer response for version 2

**DOI:** 10.21956/openreseurope.18656.r48399

**Published:** 2024-12-20

**Authors:** Andrew Geddes

**Affiliations:** 1 European University Institute, Fiesole, Italy

The article has a high level of ambition, which is very welcome. It covers a very extensive literature and is clearly written. I am aware of the guidelines for this review and will comment in a way that is consistent with the terms that are provided for reviewers.

 My overall impression is that this is a book manuscript squeezed into an article. The ground that is covered is vast, which makes it hard to follow the argument at times because so many elements of the debate are covered. For example, lots of other researchers' work is referred to, but the analysis can seem superficial because it's not possible to report all the claims that are made in the very many sources that are referred to 

 The paper advocates a humanising perspective as opposed to what it characterises as narrow more rationalist accounts of migration that cannot account for contemporary transnational migration. It also argues for a greater emphasis on temporal and psychological dimensions, including emotions and cognitive processes. I think these are good points, but the way they are presented as being new insights is hard to sustain. 

 For example, the core claim made by the article seems to me to be quite surprising. In my view, there is a large amount of work in a variety of disciplines that places the human dimension of migration at the centre of its approach with an emphasis on migrants lives, voices and experiences. It also seems to me that this article also indirectly acknowledges this because it refers to much existing research that I think does humanise migration in the terms defined by this paper. In that sense, the term humanising loses its analytical value because it contains within it a wide range of ontologies and epistemologies and can't easily be aggregated into one humanising approach.

 If the argument is about temporal and psychological dimensions then it could make sense to make these more central to the article and to strengthen the focus on these without feeling the need to cover so much ground.  

 The author might like to reflect whether the term 'humanising' is the best way to frame this analysis given that there is a lot of other research on migration that is consistent with such an approach. In such terms, perhaps it might be more feasible to not represent this as though it is a new approach, but, rather, represent the article as an attempt to synthesise and organise a wide range of material that seeks to place the experience of migrants at the core of its approach. Like this, the article is not explicitly criticising migration researchers for missing or ignoring this human angle - which is not a claim that can be substantiated given the huge amout of work that does precisely this. Rather, the article seeks to bring together work that locates and contextualises migrant agency and experiences. The paper's ambition could then be to synthesise rather than to develop a new theorisation which also has the disadvantage of seeming to suggest that researchers have somehow 'dehumanised' migrants.

 It seem to me that it could be feasible to say more about the temporal and psychological dimensions in relation to engendering and decolonising. Both the gendered  dimension of migration and colonial/neocolonial links have been and still are important parts of the debate about migration. I do see a quite strong temporal focus in much research (most obviously in decolonising work), but I would like to read more about psychology, the role of emotions etc. The references in the paper to emotions are at a more general level, but, if the aim is to build bridges with psychology, then it could perhaps be helpful to look beyond migration research and to consider more generally & with what effects emotions are activated & which emotions are activated etc.    

Is the argument persuasive and supported by appropriate evidence?

Partly

Is the work clearly and cogently presented?

Partly

Is the topic of the essay discussed accurately in the context of the current literature?

Partly

Does the essay contribute to the cultural, historical, social understanding of the field?

Partly

Reviewer Expertise:

The global poliitcs of migration, mobility and asylum

I confirm that I have read this submission and believe that I have an appropriate level of expertise to confirm that it is of an acceptable scientific standard, however I have significant reservations, as outlined above.

# Reviewer report: Reviewer response for version 2

**DOI:** 10.21956/openreseurope.18656.r40114

**Published:** 2024-05-13

**Authors:** Ulrike Schuerkens

**Affiliations:** 1 University Rennes 2, Rennes, France

The article is interesting and witnesses of a large literature review of the author. However, the article is for me more or less a short introduction of a specialized monograph that would discuss with many details the theoretical topic of migration studies. In the form presented here, the large theoretical discussion in the field doesn't appear with the details of approaches that are needed to confirm the necessity of the changes that the author wishes to see in the field of migration studies.

 I can agree to the three fields that the author suggests to modify. However, a scientific research project only turns around a small part of research questions and cannot tackle the many different methods and topics that the author wants to see in migration studies. These many research endeavors may become research projects that the author would study over the rest of her professional life as a social scientist with colleagues in the field of migration.

 Moreover, I cannot follow the author in her emphasis put on "humanising research". Migration studies in sociology and anthropology focus on humans, their agency, their fears and intentions. Perhaps this term should be replaced by another term that considers in a better way the long tradition of migration studies.

Is the argument persuasive and supported by appropriate evidence?

Partly

Is the work clearly and cogently presented?

Partly

Is the topic of the essay discussed accurately in the context of the current literature?

Partly

Does the essay contribute to the cultural, historical, social understanding of the field?

Yes

Reviewer Expertise:

Migratiion theory, migration and development, African migrations South of the Sahara, theories of social change and empirical studies.

I confirm that I have read this submission and believe that I have an appropriate level of expertise to confirm that it is of an acceptable scientific standard, however I have significant reservations, as outlined above.

# Reviewer report: Reviewer response for version 1

**DOI:** 10.21956/openreseurope.17794.r35017

**Published:** 2023-10-16

**Authors:** Saara Koikkalainen

**Affiliations:** 1 Karelian Institute, University of Eastern Finland, Joensuu, Finland

The article discusses an important and partly understudied topic: migration decision-making. It includes an excellent overview of the state-of-the-art of the field and has an extensive list of references, which serves as a great starting point for anyone interested in this topic.

 The article proposes a framework for understanding why some people in comparable situations migrate, while others do not. This the author calls "humanising research on (non-)migration decision-making".  Having the (non-) included in the name of the framework makes sense, as the author wants to highlight the fact, that some of those who think about migrating never realise their plans. However, I would stress this fact in the text of the article but would drop it from the title and name of the framework, as it makes it a bit cumbersome to read and use. 

 The author argues that there are three key aspects of research that can humanise migration research: thick contextualisation, life dimension-focused analysis, and time-situated inquiry. These are well presented and seem very valid in the pursuit of understanding migration from the individual's perspective. Taking account of these three aspects can be useful when planning new research on a particular migration phenomenon.

 The article includes five figures that are meant to clarify mainstream migration theorisations, thick contextualisation, migrant's internal processes, temporality in migration decision-making, and finally the "humanising (non-)migration decision-making framework.". Some of the figures contain too many levels and issues to really be useful for the reader. One wonders if they are all necessary? Yet the final figure does portray the key ingredients of the proposed framework in a neat and informative manner and is, therefore, a good addition to the article text. 

 One minor change that I would recommend is to edit the first sentence of the abstract as this is not the clearest or most inviting opening line: "Recent global challenges such as the COVID-19 pandemic, economic crises, and wars have not impeded transnational migration to continuously unfold." The article does not really discuss the impact of such events on migration decision-making, so I would just replace it with the opening of the plain language summary.

 In conclusion, the article is an important contribution to the field of migration research and it can be recommended for all those who wish to better understand how the individual perspective and life situation of current and future migrants influence the formation of migration patterns.

Is the argument persuasive and supported by appropriate evidence?

Yes

Is the work clearly and cogently presented?

Yes

Is the topic of the essay discussed accurately in the context of the current literature?

Yes

Does the essay contribute to the cultural, historical, social understanding of the field?

Yes

Reviewer Expertise:

Migration decision-making

I confirm that I have read this submission and believe that I have an appropriate level of expertise to confirm that it is of an acceptable scientific standard.

# Response

**Published:** 2024-02-01

**Authors:** Asuncion Fresnoza-Flot

**Affiliations:** Université libre de Bruxelles, Brussels, Belgium

Replies to the review of Saara Koikkalainen (16 October 2023): The article discusses an important and partly understudied topic: migration decision-making. It includes an excellent overview of the state-of-the-art of the field and has an extensive list of references, which serves as a great starting point for anyone interested in this topic.

 - Thank you for this positive review of the paper.  

 The article proposes a framework for understanding why some people in comparable situations migrate, while others do not. This the author calls "humanising research on (non-)migration decision-making". Having the (non-) included in the name of the framework makes sense, as the author wants to highlight the fact, that some of those who think about migrating never realise their plans. However, I would stress this fact in the text of the article but would drop it from the title and name of the framework, as it makes it a bit cumbersome to read and use.

 - Following your suggestion, I deleted the prefix “(non-)” in the title and in the name of the proposed framework.  

 The author argues that there are three key aspects of research that can humanise migration research: thick contextualisation, life dimension-focused analysis, and time-situated inquiry. These are well presented and seem very valid in the pursuit of understanding migration from the individual's perspective. Taking account of these three aspects can be useful when planning new research on a particular migration phenomenon.

 - Thank you for this validation of the three key aspects advanced in the paper.   

 The article includes five figures that are meant to clarify mainstream migration theorisations, thick contextualisation, migrant's internal processes, temporality in migration decision-making, and finally the "humanising (non-)migration decision-making framework.". Some of the figures contain too many levels and issues to really be useful for the reader. One wonders if they are all necessary? Yet the final figure does portray the key ingredients of the proposed framework in a neat and informative manner and is, therefore, a good addition to the article text.

 - Indeed, Figures 2 and 3 are partly redundant with Figure 5 as they zoom in on specific elements of this final figure. However, given that Figure 5 is quite complex, I prefer to keep Figures 2 and 3 to facilitate understanding of Figure 5.  

 One minor change that I would recommend is to edit the first sentence of the abstract as this is not the clearest or most inviting opening line: "Recent global challenges such as the COVID-19 pandemic, economic crises, and wars have not impeded transnational migration to continuously unfold." The article does not really discuss the impact of such events on migration decision-making, so I would just replace it with the opening of the plain language summary.

 - I deleted the sentence in question, but I did not move the opening of the plain language summary to the abstract, as it has the same idea as the new starting sentence of the said section.  

 In conclusion, the article is an important contribution to the field of migration research and it can be recommended for all those who wish to better understand how the individual perspective and life situation of current and future migrants influence the formation of migration patterns.

 - Thank you for this positive assessment of my article’s contribution to the field.  

 Is the topic of the essay discussed accurately in the context of the current literature? Yes

 Is the work clearly and cogently presented? Yes

 Is the argument persuasive and supported by appropriate evidence? Yes

 Does the essay contribute to the cultural, historical, social understanding of the field? Yes

 - Thank you for your constructive comments and validation of this proposed review paper to ORE.

# Reviewer report: Reviewer response for version 1

**DOI:** 10.21956/openreseurope.17794.r35014

**Published:** 2023-10-09

**Authors:** Jørgen Carling

**Affiliations:** 1 Peace Research Institute Oslo, Oslo, Norway

This paper has the ambition to ‘propose an analytical framework to address the question of what drives people to migrate, remigrate, or stay where they are.’ This is a tall ambition, and a welcome one. The development of migration research benefits from papers that address the fundamental issues of the field, as this paper does.

 However, the there are two related disjunctures with this submission. First, the scope and ambition of the paper is, at the overall level, not matched by equally thorough and precise analysis and writing. The author engages with broad swaths of the relevant literature, but it is less clear what specific contributions the paper makes. I will return to examples.

 Second, there is a bit of a mismatch between the ambition of the paper and the choice of outlet. Open-Research Europe (ORE) has a policy of publishing every submission that meets basic checks, and peer-reviewers are instructed to ‘assess the validity of the article content, rather than the novelty or interest levels’. In other words, if the article contains nothing new or interesting, it should still pass peer review as long as it is ‘scientifically valid’. This approach, is, founded on an epistemology of cumulative positivist science and, in the case of ORE, extended from biomedical disciplines to all scientific fields.

 The nature of the article makes ‘scientific validity’ a challenging yardstick for assessment. The weaknesses in this paper can not be construed as a lack of validity, so I will approve it as a peer reviewer, in line with ORE’s philosophy. In other words, there is nothing ‘wrong’ even though, in my opinion, the paper falls far short of its ambitions and is not (yet) a truly interesting or useful contribution to the field.

 (An aside for the author’s information: If I had reviewed the paper for a regular selective journal with demands for quality, novelty and interest, I would have recommended major revision. There is no prohibition against making suggestions for improvements in an ORE review. However, it is a better use of my reviewing capacity to do that for journals in which such improvements are criteria for publication. This paper apparently draws on the proposal for the AspirE project, and it might be that it has been submitted in order to create a citeable reference for this framework, without an ambition to increase its novelty or interest. This would be a fair motivation for selecting ORE as the target outlet.)

 The ORE review form poses several questions, which I reproduce here along with my answers and justification.

*Question:* Is the topic of the essay discussed accurately in the context of the current literature?

*Answer:* Partly.

 The paper cites a great deal of relevant literature. However, it does not always engage with that literature in a precise and useful way. I will give a few examples, but an exhaustive account would go far beyond the reviewer role.

 The label ‘humanising’ is problematic for two reasons. First, the human dimensions of migration decision-making have been extensively addressed by other researchers and incorporated in theorizing over the past two decades. The claim that ‘mainstream migration theories focus on individuals’ rationality to uncover the logics behind their spatial mobility, thereby neglecting other important dimensions of human lives’ might have been true 30 or 40 years ago, but the paper itself extensively cites migration theory from the past couple of decades that is both well-established and sensitive to human aspects.

 Second, the label ‘humanising’ has a somewhat self-aggrandising, moralising ring. It implies that other scholars ‘dehumanise’ migrants and/or migration decision-making. It’s never good to be inaccurate about existing research, of course, but it is worse when it is done in a moralising way. I am sure this was not the author’s intention, but it is the nevertheless the effect of the chosen framing.

 Another problematic usage appears with ‘transnational’ and ‘transnationalism’. The Introduction starts by defining the article’s scope as ‘transnational migration’. This term is avoided by many theorists, including myself. The study of ‘transnationalism’ and ‘transnational practices’ has been a key development in migration studies since the 1990s. Early on, it was noted that the word ‘transnational’ is often used loosely and inaccurately as a sign of sophistication rather than analytical specificity. Doing so creates confusion between international migration in general and the ways in which some migrants, but not all, engage in transnational practices and create transnational social fields. Later in the paper, ‘Transnationalism’ appears in the figure that displays ‘mainstream migration theorisations’. But it is a misunderstanding that ‘transnationalism’ is (or was ever intended to be) a theory of migration.

 The claim that ‘each discipline offers a migration theory suited for a specific context and situation’ and that de Haas is the only theorist that has offered an interdisciplinary approach is clearly a misleading representation. (A minor point on terminology in connection with this discussion: de Haas has developed the ‘aspirations and capabilities framework’ while Carling developed the ‘aspiration/ability model’). There is one textbook that takes a discipline-by-discipline approach, but all the major migration journals are interdisciplinary, many leading migration research environments are interdisciplinary, and many theorists draw extensively on the work of scholars trained in disciplines other than their own. The emphasis on the value of interdisciplinarity is also a bit awkward in the context of this paper, which appears completely detached from quantitative methods.

 The subsequent claims about scholars only analysing one analytical level and preferring unidimensional approaches are equally inaccurate, I think.

 Perhaps these sweeping generalizations reflect the paper’s origins in a research proposal? The pressure to obtain funding can result in exaggerated claims to novelty and dismissive blanket claims about existing research. Maybe that’s just part of the proposal genre, but I don’t think it is helpful as part of the scientific literature.

*Questions:* Is the work clearly and cogently presented? Is the argument persuasive and supported by appropriate evidence? Does the essay contribute to the cultural, historical, social understanding of the field?

*Answer:* Partly (to all three questions)

 These questions are closely related, so I address them jointly. The paper’s argument is, essentially, that the ‘humanising (non-)migration decision-making’ is a novel and useful contribution to the study of migration. I am not persuaded that it is. But, as noted above, this is irrelevant to the ORE peer review criteria.

 I am completely sympathetic to most of the claims in the paper, for instance about the value of interdisciplinarity, the importance of human dimensions beyond rationality, and the need for incorporating analyses of gender relations in theory. However, I think all these points have been made before, and in more analytical and precise ways. (Again, this is something that would have stood in the way of publication in a selective journal, but not under the publishing model of ORE.)

 The paper seeks to extract and discuss the methodological implications of the proposed theoretical framework. This is a very welcome ambition. However, it might be overly ambitious, and is, in my opinion, not very successful. The thrust of the argument on methods almost seems to be that researchers must gain an overview of everything: ‘researchers need to provide detailed information about the social world these individuals live in’ as well as giving critical attention to ‘the natural environmental contexts’ and carrying out ‘archival and other forms of documentary research, as well as content analysis of selected documents (e.g., texts of laws and policies, historical accounts, journalistic and government reports, or statistical data)’. This comes in addition to empirical data from research participants.

 In practice, methodology is also about managing financial and intellectual resources in the best possible way for generating valuable new knowledge. In-depth investigation of social worlds, natural environments, archives, legislation, and historical accounts is possible, of course, but might preclude examining a diversity of contexts, for instance, or spending more time in the field with research participants. In short, ‘more is better’ is not very useful as methodological advice.

 A more specific point in the discussion concerns participatory methods. Again, this is a valuable part of a holistic approach. However, the value of the discussion is limited by not problematizing key issues such as who should participate and what is the value for participants of contributing their time. It is also a concern, I think, that the text refers to ‘the participatory technique called “focus group discussion”’. Focus group discussions are a widely used data collection format in migration study, but it is not inherently participatory.

 In conclusion, I value the ambition of this paper and sympathize with much of its spirit, but believe that the quality, novelty and usefulness of the text itself do not match. Submission to a selective journal that apply such criteria would have resulted in an uphill struggle that might have ended with a much improved paper. The choice of ORE is a legitimate alternative strategy for making the text available and citeable. It is up to the author what more to aim for.

Is the argument persuasive and supported by appropriate evidence?

Partly

Is the work clearly and cogently presented?

Partly

Is the topic of the essay discussed accurately in the context of the current literature?

Partly

Does the essay contribute to the cultural, historical, social understanding of the field?

Partly

Reviewer Expertise:

Migration theory; migration decision-making; migration research methods; interdisciplinary research.

I confirm that I have read this submission and believe that I have an appropriate level of expertise to confirm that it is of an acceptable scientific standard, however I have significant reservations, as outlined above.

# Response

**Published:** 2024-02-01

**Authors:** Asuncion Fresnoza-Flot

**Affiliations:** Université libre de Bruxelles, Brussels, Belgium

*Replies to the review of Jørgen Carling (09 October 2023):* This paper has the ambition to ‘propose an analytical framework to address the question of what drives people to migrate, remigrate, or stay where they are.’ This is a tall ambition, and a welcome one. The development of migration research benefits from papers that address the fundamental issues of the field, as this paper does.

 - Thank you for welcoming this effort to contribute to the development of migration research.  

 However, the there are two related disjunctures with this submission. First, the scope and ambition of the paper is, at the overall level, not matched by equally thorough and precise analysis and writing. The author engages with broad swaths of the relevant literature, but it is less clear what specific contributions the paper makes. I will return to examples.

 - I added an explanation in paragraphs 2 and 3 of the Conclusion section to clarify the paper’s contributions. First, I indicated that the proposed framework in the paper framework can “provide more nuances in the way (non-)migration decision-making has been understood, thereby avoiding generalising discourses regarding transnational migrations”. And second, I explained that the paper’s proposed humanising methodologies can “provide rich contextual and empirical data, which can further clarify the whole picture of (non-)migration decision-making and therefore reduce the risk of a biased portrayal of the said process”.  

 Second, there is a bit of a mismatch between the ambition of the paper and the choice of outlet. Open-Research Europe (ORE) has a policy of publishing every submission that meets basic checks, and peer-reviewers are instructed to ‘assess the validity of the article content, rather than the novelty or interest levels’. In other words, if the article contains nothing new or interesting, it should still pass peer review as long as it is ‘scientifically valid’. This approach, is, founded on an epistemology of cumulative positivist science and, in the case of ORE, extended from biomedical disciplines to all scientific fields.

 - My choice of ORE as platform to publish the proposed paper stems from three major reasons. First, it is aligned with the Open Science principle that the project in which the paper is inscribed intends to pursue. I was not aware of ORE’s existence until the project started and I received an invitation to publish on the said platform. As the project is funded by the European Commission’s Horizon Europe programme, I decided to submit my article there to support the Commission’s ORE initiative. Second, since ORE attracts researchers from other disciplines, publishing a paper on its platform was a way for me to communicate the project’s starting analytical framework to a wide, multidisciplinary readership. And third, my paper appeared well fitted to the criteria in the guidelines to authors of ORE according to which a review article should “provide a balanced and comprehensive overview of the latest discoveries in a social science discipline and summarize topics that have not yet been covered in the same way in the existing published literature. Reviews should be based on peer-reviewed literature, and should not include new research, data or propose new hypotheses” ( https://open-research-europe.ec.europa.eu/for-authors/article-guidelines/social-sciences/reviews). These criteria seem at odd with the instructions to reviewers you mention (which I discovered at https://open-research-europe.ec.europa.eu/for-referees/guidelines after are reading your review comments),  according to which reviewers should “assess the validity of the article content, rather than the novelty or interest levels” of a paper. In my opinion, this instruction clearly does not apply to review papers since they do not contain any new data. I believe that ORE should clarify their instructions to reviewers to bring them in line to their guidelines to authors regarding review papers.  

 The nature of the article makes ‘scientific validity’ a challenging yardstick for assessment. The weaknesses in this paper can not be construed as a lack of validity, so I will approve it as a peer reviewer, in line with ORE’s philosophy. In other words, there is nothing ‘wrong’ even though, in my opinion, the paper falls far short of its ambitions and is not (yet) a truly interesting or useful contribution to the field.

 - Thank you for accepting to review the paper despite its limitations. I revised my article paper to address your comments and suggestions by highlighting its possible contributions to the research field on decision-making of aspiring (re)migrants. Given its very nature as a review essay, the paper’s “truly interesting or useful contribution to the field” may not be immediately evident and may only come out once its proposed analytical framework is applied to empirical research. An empirically based paper using the said framework will be submitted for publication at the end of the project in which this review essay is inscribed.   

 (An aside for the author’s information: If I had reviewed the paper for a regular selective journal with demands for quality, novelty and interest, I would have recommended major revision. There is no prohibition against making suggestions for improvements in an ORE review. However, it is a better use of my reviewing capacity to do that for journals in which such improvements are criteria for publication. This paper apparently draws on the proposal for the AspirE project, and it might be that it has been submitted in order to create a citeable reference for this framework, without an ambition to increase its novelty or interest. This would be a fair motivation for selecting ORE as the target outlet.)

 - The paper is a review essay that unveils the conclusions of an evaluation of the literature on migration theorisations. Its ambition is to share this evaluation to the academic community at large and to receive feedback from experts in order to further improve the analytical framework proposed in the paper. Once validated by experts, this framework will serve as a starting analytical optic through which the empirical data in the AspirE project will be examined and interpreted.  

 The ORE review form poses several questions, which I reproduce here along with my answers and justification.

*Question*: Is the topic of the essay discussed accurately in the context of the current literature?

*Answer*: Partly.

 The paper cites a great deal of relevant literature. However, it does not always engage with that literature in a precise and useful way. I will give a few examples, but an exhaustive account would go far beyond the reviewer role.

 - I hope that the revised version of the paper does not convey anymore this impression.  

 The label ‘humanising’ is problematic for two reasons. First, the human dimensions of migration decision-making have been extensively addressed by other researchers and incorporated in theorizing over the past two decades.

 -  In the last paragraph of the state-of-the-art section I added a sentence to emphasise that “several theories in the past three decades have extensively addressed the human dimensions of migration”. In the previously submitted version of the paper, paragraph 3 in the state-of-the-art section already brought to the fore the contributions of gender and migration research in highlighting the relational aspects of migration through the gender lens. In paragraph 4 of the same section of the paper, it mentioned that “certain theories and empirical works have pointed out how imaginaries and emotions affect an individual’s decision-making process”, but that despite this development “the internal processes (cognitive and emotional) of individuals remain rarely treated together in the analysis”. The previously submitted version also explained that “scholars increasingly criticise the neglect of the temporal dimension in the analysis of migration”. To emphasise further these past efforts, I added a summary in the last paragraph of the state-of-the-art section in the revised paper stating that “not all dimensions are taken into account” in several theories of the past three decades, “notably internal processes (emotional and cognitive), time (stages and timing), and individual’s social locations “within interconnected power hierarchies”.  

 The claim that ‘mainstream migration theories focus on individuals’ rationality to uncover the logics behind their spatial mobility, thereby neglecting other important dimensions of human lives’ might have been true 30 or 40 years ago, but the paper itself extensively cites migration theory from the past couple of decades that is both well-established and sensitive to human aspects.

 -  To better align the introduction of the paper with the state-of-the-art section that cited migration theories from the past couple of decades, I added a sentence stating that the mainstream migration theories referred to concern “notably classical” theories. I also added in paragraph 2 of the introduction two sentences summarising one of the key observations presented in the state-of-the-art section: “More and more migration theories in the past three decades take into account the different dimensions of human lives, such as in relational, experiential, and perceptual terms. Nonetheless, the temporal and psychological dimensions including emotions, as well as other cognitive processes, remain largely overlooked as recent works point out”.  

 Second, the label ‘humanising’ has a somewhat self-aggrandising, moralising ring. It implies that other scholars ‘dehumanise’ migrants and/or migration decision-making. It’s never good to be inaccurate about existing research, of course, but it is worse when it is done in a moralising way. I am sure this was not the author’s intention, but it is the nevertheless the effect of the chosen framing.

 - I clarified in the revised version of the paper the use of the qualifier “humanising” (see paragraph 2 of the introduction) to avoid giving the impression of implying that other scholars “dehumanise” migrants and/or migration decision-making. I explained that the adjective “humanising” is used in my paper to depart from the qualifiers “humanist” and “humanistic” that  “have acquired problematic connotations due to their links to humanism and humanistic psychology, respectively”. Taking into account Kerr’s (2007) definition of “humanising’ as ‘making better’ or ‘improving’” (6), the use of the said qualifier in my paper may avoid “dichotomic thinking in terms of good and bad or humane and inhumane”. I also find “humanising” well “appropriate to employ when the focus includes individual’s voices and experiences”.  

 Another problematic usage appears with ‘transnational’ and ‘transnationalism’. The Introduction starts by defining the article’s scope as ‘transnational migration’. This term is avoided by many theorists, including myself. The study of ‘transnationalism’ and ‘transnational practices’ has been a key development in migration studies since the 1990s. Early on, it was noted that the word ‘transnational’ is often used loosely and inaccurately as a sign of sophistication rather than analytical specificity. Doing so creates confusion between international migration in general and the ways in which some migrants, but not all, engage in transnational practices and create transnational social fields.

 - I added in paragraph 1 of the introduction section an explanation about the logic behind the use of the term “transnational migration” in the paper. Drawing from Portes (2001, p. 186), I used the qualifier “transnational” to describe the geographic movements of people across national borders for two reasons: first, it signifies that the migrations I am referring to frequently stem from non-institutional actors’ initiatives and sustained efforts “across national borders”, and second, it indicates that they take place most often “outside the pale of state regulation and control”. This choice of the qualifier “transnational” rather than “international” allows me to emphasise the “informal” dimension or the “bottom” aspect of (non-)migration decision-making.  

 Later in the paper, ‘Transnationalism’ appears in the figure that displays ‘mainstream migration theorisations’. But it is a misunderstanding that ‘transnationalism’ is (or was ever intended to be) a theory of migration.

 - This mistake in Figure 1 has now been corrected, thanks for pointing it out.  

 The claim that ‘each discipline offers a migration theory suited for a specific context and situation’ and that de Haas is the only theorist that has offered an interdisciplinary approach is clearly a misleading representation.

 - I revised this whole paragraph to clarify what I meant. The paper does recognise that interdisciplinarity has been present in migration theorisation, but there is a need for more interdisciplinarity as some dimensions of human lives (e.g., temporality and psychological aspects) receive limited attention in existing theories.  

 (A minor point on terminology in connection with this discussion: de Haas has developed the ‘aspirations and capabilities framework’ while Carling developed the ‘aspiration/ability model’). - Thank you for bringing this point to my attention, it is now corrected in the revised version of my paper.  

 There is one textbook that takes a discipline-by-discipline approach, but all the major migration journals are interdisciplinary, many leading migration research environments are interdisciplinary, and many theorists draw extensively on the work of scholars trained in disciplines other than their own.

 - I agree that “all the major migration journals are interdisciplinary, many leading migration research environments are interdisciplinary”, but this interdisciplinarity is not generally the case in terms of migration theorisation as the state of the art in the paper shows. That is why I highlight in the last paragraph of the state-of-the-art section “the need for migration theorisation to be more interdisciplinary”. I also agree that “many theorists draw extensively on the work of scholars trained in disciplines other than their own”, but as I explained in the paper, the resulting theories mostly “remain within their respective disciplines” or fields of studies. In my opinion, this situation is not “interdisciplinarity” (i.e., “the reciprocal interaction between […] disciplines […] in order to generate new common methodologies, perspectives, knowledge, or even new disciplines”: Choi & Pak, 2006, 359) but rather “multidisciplinarity” in which scholars draw from different disciplines but remain within their “disciplinary boundaries” (ibid.).  I added supporting references in the last paragraph of the state-of-the-art section, explaining that recent scholarly works observe the compartmentalisation in migration studies itself and underlines the need for more interdisciplinarity. I gave the following examples: in gender and migration research, Hondagneu-Sotelo (2011) observes that researchers in different spheres of studies “are mostly not in conversation with one another” due “to the increasingly specialized and balkanized nature of social science research today” (277). In the research field of migration and integration, Dahinden (2016) proposes “to link migration research issues more closely with social science and theory in general” as one of several strategies to “de-migrantize” the said field (2214). I also cited Koikkalainen and Kyle’s (2016) observation about the need for “social psychology or cognitive social sciences” to be incorporated in migration studies, in the study of (non-)migration decision-making.  

 The emphasis on the value of interdisciplinarity is also a bit awkward in the context of this paper, which appears completely detached from quantitative methods.

 - Since the paper proposes a decolonising methodology for a study of migration decision-making, it privileges qualitative and participatory data collection methods that bring to the fore the “emic perspectives (i.e., individual’s points of view) and diversity of voices, discourses, and experiences”. This choice does not completely exclude quantitative methods, but qualitative and participatory methodologies appear sufficiently effective to access what Tilly (2010) calls “orally transmitted knowledge” central to the “decolonising” stance adopted in my article. I added this point in paragraph 1 in the section “Humanising methodologies”.  

 The subsequent claims about scholars only analysing one analytical level and preferring unidimensional approaches are equally inaccurate, I think.

 - The paper does not claim that scholars only analyse one analytical level. What it explains is that what has “been widely adopted in migration studies” is macro-level migration theories and that theories with “meso- and micro-level frameworks, specifically their combination and articulation with macro-level lenses, remain scarce” (see paragraph 5 of the state of the art). I clarified in the last paragraph of the state-of-the-art section that classical migration theories tend to focus on one level of analysis (the macro level). Concerning unidimensional approach, I provided examples to strengthen the observation I laid out in the last paragraph of the state-of-the-art section.  

 Perhaps these sweeping generalizations reflect the paper’s origins in a research proposal? The pressure to obtain funding can result in exaggerated claims to novelty and dismissive blanket claims about existing research. Maybe that’s just part of the proposal genre, but I don’t think it is helpful as part of the scientific literature.

 - The paper does not intend to generalise beyond the corpus of scholarly works it reviewed (listed in the References section). There are certainly works written in English and other languages that escaped the analytical gaze of the paper, which is one of the limitations that I indicated in the revised conclusion of the paper.  

*Questions*: Is the work clearly and cogently presented? Is the argument persuasive and supported by appropriate evidence? Does the essay contribute to the cultural, historical, social understanding of the field?

*Answer*: Partly (to all three questions) These questions are closely related, so I address them jointly. The paper’s argument is, essentially, that the ‘humanising (non-)migration decision-making’ is a novel and useful contribution to the study of migration. I am not persuaded that it is. But, as noted above, this is irrelevant to the ORE peer review criteria.

 - The paper proposes a framework that has not been applied yet to any empirical studies, hence it is difficult to assess its concrete contributions to the research field of migration decision-making and/or migration per se. I included this point in the discussion of the paper’s limitations in the last paragraph of the conclusion section. In the meantime, the framework proposed in the paper based on a literature review is opened to be discussed and criticised for its further development.  

 I am completely sympathetic to most of the claims in the paper, for instance about the value of interdisciplinarity, the importance of human dimensions beyond rationality, and the need for incorporating analyses of gender relations in theory. However, I think all these points have been made before, and in more analytical and precise ways. (Again, this is something that would have stood in the way of publication in a selective journal, but not under the publishing model of ORE.)

 - Yes, the aforementioned points have been made before, but they have been raised separately in several research fields and studies. What the paper proposed is to heed and take into account these separate calls into one framework so as not to overlook all aspects/dimensions of human life in the analysis of (non-)migration decision-making.  

 The paper seeks to extract and discuss the methodological implications of the proposed theoretical framework. This is a very welcome ambition. However, it might be overly ambitious, and is, in my opinion, not very successful. The thrust of the argument on methods almost seems to be that researchers must gain an overview of everything: ‘researchers need to provide detailed information about the social world these individuals live in’ as well as giving critical attention to ‘the natural environmental contexts’ and carrying out ‘archival and other forms of documentary research, as well as content analysis of selected documents (e.g., texts of laws and policies, historical accounts, journalistic and government reports, or statistical data)’. This comes in addition to empirical data from research participants.

 - The paper does not intend to convey that “researchers must gain an overview of everything”. To clarify this point, I added an explanation in paragraph 2 of the section “Humanising methodologies” that the examples of data collection techniques provided in the said section “represent a pool of methodological possibilities from which researchers can choose the most appropriate data-collection technique(s) for their respective studies”. As already stated in its introduction section, the paper only “suggests some methodological directions” in how to pursue the analytical framework it proposes.  

 In practice, methodology is also about managing financial and intellectual resources in the best possible way for generating valuable new knowledge. In-depth investigation of social worlds, natural environments, archives, legislation, and historical accounts is possible, of course, but might preclude examining a diversity of contexts, for instance, or spending more time in the field with research participants. In short, ‘more is better’ is not very useful as methodological advice.

 - I agree that “‘more is better’ is not very useful as methodological device” (cf. my reply to the previous comment). That is why the paper specifically highlights the value of qualitative and participatory methods and does not include quantitative methods. It does not completely exclude the possibility of adding the latter to the former but argues that for a framework with a “decolonising” stance qualitative and participatory methodologies appear sufficiently effective to access “orally transmitted knowledge” (Tilly, 2010). I added this point in paragraph 1 in the section “Humanising methodologies”.  

 A more specific point in the discussion concerns participatory methods. Again, this is a valuable part of a holistic approach. However, the value of the discussion is limited by not problematizing key issues such as who should participate and what is the value for participants of contributing their times.

 - Thank you for raising this point that I took into account in the revised version of the paper, specifically in paragraph 3 of the section “Humanising methodologies”. I explained in this paragraph that “(t)o reinforce the decolonising aspect of focus group discussion, researchers should take into account its ethical dimension. For example, there is a need for a transparent research protocol and a thorough reflection on how to address the questions of who will be invited to take part in the discussion, how their participation be compensated, or how their personal data will be protected”.  

 It is also a concern, I think, that the text refers to ‘the participatory technique called “focus group discussion”’. Focus group discussions are a widely used data collection format in migration study, but it is not inherently participatory.

 - I agree with your observation that “(f)ocus group discussions are a widely used data collection format in migration study, but it is not inherently participatory”. That is why the section “Humanising methodologies” of my paper draws from outside of migration studies, specifically from psychology and sociology, where focus group discussions have been used as one form of participatory methods. I provided supplementary information about this use of focus group discussion in paragraph 4 of the section “Humanising methodologies”.  

 In conclusion, I value the ambition of this paper and sympathize with much of its spirit, but believe that the quality, novelty and usefulness of the text itself do not match. Submission to a selective journal that apply such criteria would have resulted in an uphill struggle that might have ended with a much improved paper.

 - This comment seems to imply that my paper could only be improved by sending it to another journal (not ORE). If this is what you mean, withdrawing my paper at this stage to resubmit it somewhere else would be difficult as I already morally engaged myself with ORE. However, I will propose the empirically based follow-up paper of this review essay to a traditional peer-reviewed journal.  

 The choice of ORE is a legitimate alternative strategy for making the text available and citeable. It is up to the author what more to aim for.

 - My choice of ORE was neither to escape rigorous peer review nor to use it as a mere “strategy for making the text available and citeable”. As explained at the beginning of my replies, the choice stemmed from the commitment of my project to Open Science, to my intent of sharing the project’s analytical framework with a wide readership across disciplines, and to the fact that my review paper seems to correspond well to the criteria set forth by ORE for a review article in a social science discipline. My aim is therefore to improve this review paper, and I hope that its revised version will now match your standards.
